# Termites utilise clay to build structural supports and so increase foraging resources

**DOI:** 10.1038/srep20990

**Published:** 2016-02-08

**Authors:** Sebastian Oberst, Joseph C. S. Lai, Theodore A. Evans

**Affiliations:** 1 Acoustics & Vibration Unit, School of Engineering and Information Technology, The University of New South Wales, ADFA, Canberra, ACT 2600, Australia; 2 CSIRO Ecosystem Sciences, Clunies Ross Street, Canberra ACT 2600, Australia; 3 School of Animal Biology, University of Western Australia, Perth, WA, 6009, Australia

## Abstract

Many termite species use clay to build foraging galleries and mound-nests. In some cases clay is placed within excavations of their wooden food, such as living trees or timber in buildings; however the purpose for this clay is unclear. We tested the hypotheses that termites can identify load bearing wood, and that they use clay to provide mechanical support of the load and thus allow them to eat the wood. In field and laboratory experiments, we show that the lower termite *Coptotermes acinaciformis*, the most basal species to build a mound-nest, can distinguish unloaded from loaded wood, and use clay differently when eating each type. The termites target unloaded wood preferentially, and use thin clay sheeting to camouflage themselves while eating the unloaded wood. The termites attack loaded wood secondarily, and build thick, load-bearing clay walls when they do. The termites add clay and build thicker walls as the load-bearing wood is consumed. The use of clay to support wood under load unlocks otherwise unavailable food resources. This behaviour may represent an evolutionary step from foraging behaviour to nest building in lower termites.

Cellulose is one of the most abundant biological compounds on the planet; it is most commonly found in wood, providing an enormous resource for those organisms able to digest it^1^,^2^,^3^. Termites are among the few organisms able to digest cellulose, contributing to their evolutionary and ecological success^4^,^5^. Large amounts of cellulose are found on the ground, such as logs created from fallen, dead branches, and leaf litter with even larger amounts of cellulose in living plants, such as in standing trees^6^,^7^,^8^. Some of this cellulose is under load, especially in at ground level, where subterranean termites are found. Therefore eating a log or standing tree at ground level would lead to weakening of the support and its collapse^9^, with the outcomes of death by either crushing or exposure of any surviving termites to predators^10^. Consequently, some of the most abundant cellulose resources do not appear to be available for consumption.

Human buildings may offer a parallel situation to termites, as termites seem to avoid wood under load. Anecdotally at least, termites infestations in houses are often found unexpectedly due to a ‘foot through a floor board’; i.e. termite activity is typically discovered in areas with no or low activity, in decorative or non-structural timbers, (window frames, skirting boards, door frames, or floor boards covered by or behind furniture), at least early in an infestation^10^,^11^. Termite activity in load-bearing timbers (bearers, joists, floor boards in traffic areas) appear to manifest later in infestations, and the hollows in these partially consumed timbers are often filled with clay^9^,^10^ (Fig. 1 and S1).

Neither biologists nor pest-control operators have considered the appearance of clay in hollows to be surprising, because termites are considered ‘master builders’ among insects^12^. They apply a composite of clay, saliva and faeces for a range of constructions, from simple and temporary above ground galleries (narrow tubes or broad sheeting) used to camouflage foragers, to elaborate and long-lived mounds containing their nests, which appears to be the most derived nesting behaviour, found almost entirely in the most derived termite family^13^,^14^,^15^. In all of these examples, the clay functions as building material. The clay found inside hollowed wood does not appear to serve any purpose, other perhaps than a dumping ground for soil removed by excavation during tunnelling activities.

Moving clay is energetically demanding, therefore the behaviour should be selected against, unless there is an adaptive benefit. Therefore we hypothesised that termites are building with clay in all circumstances, and that clay in hollowed timber is related to that same foraging behaviour. Termites perceive a lot of their environment using vibrational information. Termite soldiers use acoustic or vibration signals to alert colony members to danger^16^,^17^. The workers assess wood size and material properties^18^,^19^,^20^, locate their positions^20^, and recognise their own and other termite species^20^,^21^, all through vibrations. As materials under load have altered vibration characteristics^22^,^23^, we hypothesised that termites are able to assess wood load, such as wood in standing trees; then use clay to support any load, and thereby allow the wood to be eaten.

We devised two experiments to test this hypothesis. We used *Coptotermes acinaciformis* (Infraorder Isoptera, family Rhinotermitidae), a wood-eating, lower termite, which is found across mainland Australia^17^. It attacks dead wood on the ground and living *Eucalyptus* trees, and it uses clay for constructions, including within the tree trunks and a mound nest (usually abutting a hollow tree, Fig. 1a) in the tropical northern part of its range. Also, it is a major pest species; and it attacks timbers used in housing, from non-loading bearing decorative timbers to load bearing structural timbers, and it will place clay in hollows of this wood^9^,^10^ (Fig. 1b and S1).

We designed bioassays as choice experiments in the field (Fig. 2a,b) and in the laboratory (Fig. 2c,d) by applying the same principle of paired replicate units. The paired replicate units contained wooden blocks with ‘No load’, which served as the control, and wooden blocks ‘With load’, which served as the treatment. The load in the field experiment was about 245 kg and in the laboratory experiment about 1.6 kg; the wooden blocks were sandwiched between concrete pavers. The load in the controls was carried by aluminium tubing ‘spacers’, cut to the same height as the wooden blocks. In the field experiment, we aimed to mimic wood exposed to larger loads, comparable to that of standing trees, and we measured the total wood consumption and building activities by colonies over one year. In the laboratory experiment, we investigated the dynamic wood consumption and clay building behaviours of the termites under small scale and carefully controlled conditions over four weeks.

## Results

### General patterns

We found clear differences in the clay structures built by termites on ‘No load’ and ‘With load’ units in both the field and in the laboratory experiments and the differences matched in the two experiments. Termites built two types of clay structure that reached from lower to upper pavers, and so functioned as a partition. The first type of structure was thin (<1 mm) and brittle, which we categorised as ‘sheeting’, because it was similar to the well-known clay galleries and sheeting used to protect termite foraging pathways. This sheeting was so thin and brittle structure that it was incapable of supporting any load; slight pressure from a finger would reduce it to rubble. The second type of structure was greater than 10 mm thick and strong, which we categorised as ‘walls’, because it was similar to the clay walls of termite mounds.

### Field Experiment

In the field experiment 13 of the 18 replicate units had termite contact to both the ‘No load’ and ‘With load’ units, and so were included in the analyses. For the ‘No load’ units (Fig. 3a), the termites built generally thin and brittle clay sheeting, with some short, thicker and wall-like sections, most of which were under the pavers. The clay structures were built around the wooden blocks in about half of the replicates (6/13 units), or around the load bearing spacers in about a quarter of the replicates (3/13 units). The structures could extend to short walls between the two pavers (5/13 units), or extend to the outer edge of the pavers (5/13 units). On the ‘With load’ units (Fig. 3b), the termites built thick and strong clay walls in all replicates, and these thick walls were around the edge of the pavers in all 13 replicates. When the termites did build thin sheeting, it was around the wooden blocks, and rarely did the sheeting extend around the entire block (3/13 units).

In the field experiment, the ‘No load’ units had a total of 2.74 ± 2.17 kg (mean ± standard deviation) of clay, whereas the ‘With load’ units had 4.66 ± 3.30 kg of clay; a significant difference (one way analysis of variance (ANOVA), *F* = 4.74, *df* = 25, *p* = 0.0395). This difference was increased when considering the quantity of clay per unit volume between the pavers. The volume between the pavers in the ‘No load’ units remained unchanged over the experiment, due to the spacers (12,602 ± 0.02 cm^3^), whereas it was reduced by half in the ‘With load’ units (from 12,840 ± 205 cm^3^ to 6,903 ± 3,681 cm^3^); there was a highly significantly difference in clay used per unit volume (ANOVA, *F* = 32.52, *df* = 25, *p* < 0.001) (Fig. 4a).

The termites ate an average of 92% of the wood in the field experiment, perhaps unsurprising as the experiment used entire colonies and ran for one year. Initially, there was 432.27 ± 7.47 g wood in each unit. There was 14.95 ± 10.89 g wood left over in the ‘No load’ units, and 39.22 ± 62.19 g wood in the With load’ units. This was a significant difference (ANOVA, *F* = 6.20, *df* = 25, *p* = 0.0201). Normalising the quantity of wood eaten to reduce variation increased the significant difference (ANOVA, *F* = 22.53, df = 25, *p* < 0.001).

### Laboratory experiment

In the laboratory experiment 21 of the 32 replicates had contact to both units and were included in the analyses. For the ‘No load’ units (Fig. 3c), the termites built either brittle and disconnected sheeting or small heaps (diameter about 25 mm) of clay in a little over half the replicates (12/21 units), and these clay structures were concentrated around the wood pieces and spacers. The termites built no clay structures at all in a little fewer than half the replicates (9/21 units). The termites ate the wood without any clay structure reaching lower to upper pavers in one seventh of replicates (3/21 units). For the ‘With load’ units (Fig. 3d), termites built thick and solid clay walls in all replicates. These walls were concentrated at the edges of the pavers, often partly – but rarely completely – encircling the paver.

In the lab experiment, the ‘No load’ units had a total of 48.50 ± 46.81 g of clay, whereas the ‘With load’ units had 58.9 ± 43.2 g of clay, which was not significantly different (ANOVA, *F* = 0.54, *df* = 39, *p* = 0.4649). However, considering the quantity of clay per unit volume, the difference increased. The volume between the pavers in the ‘No load’ units remained unchanged over the experiments (200.50 ± 0.02 SE cm^3^), whereas it was reduced by over 80% in the ‘With load’ units (from 207.21 ± 5.03 cm^3^ to 34.15 ± 5.32 cm^3^), and the clay per unit volume differed significantly (ANOVA, *F* = 9.42, *df* = 39, *p* = 0.0039) (Fig. 4b).

The termites ate 66–86% of the wood in the laboratory experiment, even though the experiment ran for just four weeks. Initially, there was 3.44 ± 0.51 g in each unit. There was 0.49 ± 0.49 g wood remaining in the ‘No load’ units, and 1.15 ± 0.72 g wood leftover in the ‘With load’ units. This was not a significant difference (ANOVA, *F* = 1.12, *df* = 39, *p* < 0.29), however normalising the quantity of wood eaten found a significant difference (*F* = 7.05, *df* = 39, *p* = 0.0115).

### Clay substitution of wood

Termites may have substituted clay for the wood under load in a functional sense, with the clay providing the support of the load. If so, then there ought to be a negative correlation between the amounts of clay and left over wood in the ‘With load’ units and no relationship between these variables in the ‘No load’ units. To test this necessary condition we determined the distributions of leftover wood and those of clay per unit volume, and then calculated a predictive bi-variate joint beta-distribution of percentage clay per unit volume and leftover wood. We tested the data from the field and the laboratory experiments and found that they shared the same distribution (Kolmogoroff-Smirnoff *k-s* = 0.08, *p* = 0.713), and both were normally distributed (Lilliefors test field *p* = 0.354, laboratory *p* = 0.388). We fitted the relative amount of clay and the leftover wood data to marginal beta-distributions. The distributions were normalised to percentage values and hence became scale-invariant on the interval zero to one hundred (Fig. 5 and Supplementary Information). Finally, we bootstrapped 10,000 random samples of the fitted and statistically smoothed distributions^24^,^25^.

For the field and the laboratory experiments, the results were qualitatively the same: the relative amount of clay per unit volume and the leftover wood from the ‘With load’ units were inversely related. This indicated that termites substituted wood with clay progressively. The bi-variate joint distribution of the ‘With load’ units was more platykurtic than that of the ‘No load’ units, especially for the field experiment (Fig. 5), which suggested that the factors controlling the clay-wood relationship were more important over one year relative to four weeks. This may explain why the plots did not show a clear inverse ‘diagonal pattern’ as expected if simply negatively linearly correlated. The spread of the joint distribution of the laboratory experiment was larger than that of the field experiment, which indicated that an anti-proportional relation between clay per volume and left over wood existed, but that time-related processes influenced the results.

We tested directly the hypothesis that termites were substituting wood with clay, in other words that clay building would be conditional on wood consumption, using a beta-binomial model in a Bayesian updating process. This basic statistical model is similar to that of a stochastic Polya urn model as an inverse statistical model to the model of “sampling without replacement”^26^ (see Supplementary Information). The Bayesian updating had the advantage that existing information (e.g. from the past) can be included in the calculation, and that a convergence with few samples can be reached by taking the *i*^th^ posterior as the (*i* + 1)^th^ prior probability^27^.

First, we identified replicates with greater than the average amount of clay and less than fifty per cent of the initial wood in the ‘With load’ units as a ‘success’. Second, we visualized a Bayesian updated sequence of successes of replicated pairs using a beta-binomial distribution on conditional probabilities including credibility (i.e. Bayesian confidence) intervals obtained from the beta-binomial model using a uniform distribution as Jeffrey’s prior^27^, Third, we calculated the unknown median probability *p* as parameter of a binomial model using a beta-binomial model; the credible interval as highest density region (HDR) provided a 99% Credibility Interval^27^ plotted as inserts (Fig. 6). As the number of ‘successes’ accumulated in the sequence, the narrower the median probability distributions became, eventually culminating in a 99% estimated probability that termites substitute wood by clay (99% CI = 79–100%; Fig. 6a) for the field experiment, and 79% (99% CI = 60–94%; Fig. 6b) for the laboratory experiment. The wider 99% CI for the laboratory experiment is a reflection of the incomplete consumption of the wood due to a shorter experimental time period and non-ideal conditions reflected by the laboratory environment (cf. Fig. 5).

### Foraging as a dynamic process

The process of substituting wood with clay may be dynamic over time; to test this we analysed the termite activity monitored daily throughout the laboratory experiment. We identified two levels of foraging activity: ‘no contact’ when no termites were visible anywhere on the wooden block; and ‘full contact’ when termites were visible on at least one side of the wooden block. We found three distinct periods of termite activity during the 30 days of the laboratory experiment (Fig. 7). The first period appeared to be for exploration and food assessment, when similar frequencies of ‘full contacts’ were found in the ‘No load’ and ‘With load’ units. The first period lasted for an average 1.3 days (range one to three days). The second period was feeding on the wood in the ‘No load’ units, when a higher frequency of ‘full contacts’ was observed in the ‘No load’ units. Termites observed in the ‘With Load’ units did not build with clay during this period. The second period started after an average of 3.6 days (range two to eight days). The third period was feeding on the wood in the ‘With load’ units, when frequency of ‘full contacts’ increased in the ‘With load’ units, and construction of thick load supporting clay walls commenced. The third period started after an average of 19.6 days (range ten to 22 days).

## Discussion

The results of this study strongly suggested that termites were capable of distinguishing wood without load from wood with load. Termites demonstrated their capability to detect load in several ways. In the field experiment, the termites built thick clay walls in the ‘With Load’ units, using about four times more clay per volume than in the ‘No Load’ units. Similarly, in the laboratory experiment, the termites used about seven times more clay per volume in the ‘No Load’ units. Also in the laboratory experiment, the termites explored the ‘With Load’ units and the ‘No Load’ units simultaneously in the first days, but then quickly recruited to the ‘No Load’ units and ate the wood while ignoring the ‘With Load’ units. The termites only started to eat the wood in the ‘With Load’ units once they have mostly eaten the ‘No Load’ wood.

This is the first time that the capacity of termites to identify wood under load has been demonstrated. There is considerable research on how termites differentiate types of wood, based on chemical (such as flavonoids, phenolics, quinones and terpenoids) and physical (usually density, which is related to the amount of lignin within the wood)^28^,^29^,^30^,^31^. The mechanisms for detecting chemical and physical hardness are unlikely to be the same as for detecting wood under load, because the wood we used was all the same species, cut from the same pieces of lumber and thus as similar to each other as is possible to make^32^ (see Methods). Instead, the mechanisms are likely related to perception of vibrational information. Materials under load or tension have altered vibration characteristics^22^,^23^, and termites use acoustic and vibration signals for many cognitive tasks, including communication^16^,^17^, assessing wood^18^,^19^,^20^, and other species^20^,^21^.

The exact mechanics of clay structures supporting load in natural situations is unclear. In general, subterranean termites, such as *C. acinaciformis*, eat wooden objects from the ground up, whether logs, trees or housing timbers^9^,^10^,^11^. The role of clay in providing load support as shown in this study would appear to be possible in such circumstances. We tested the compressive strength of the thick clay walls using three samples from field experiments using the same mound-colonies of *C. acinaciformis* from Darwin. We found that the average compressive strength was about 0.22 MPa, which was more than enough to support the 245 kg load imposed on four wood blocks in the field trial. *Coptotermes* species eat and so excavate trees from the roots upward^9^,^10^, and thereby create a hollow cylinder. A hollow cylinder has higher flexural rigidity i.e. bending stiffness (*EI*, with *E* being the Young’s modulus and *I* the moment of inertia) than a solid one of the same mass and this increases further with larger hollows (i.e. greater inner diameter)^34^. However, filling e.g. a steel tube with concrete results in even higher stiffness and strength values until failure and higher buckling margins^34^,^35^; perhaps adding clay has a similar effect in trees.

Increasing strength may help to explain why the termites built their clay structures in various positions during the experiments. Most of the thin and brittle sheeting were built around the wood in the ‘No Load’ units, whereas most of the thick and strong walls were built around the edge of the paver, although there were exceptions. The reasons for these patterns are unknown. The normal termite foraging, galleries and sheeting are built to serve partly as camouflage, to avoid predators, and to reduce desiccation from open-air exposure. Normal foraging behaviour may be the reason the sheeting was built around the wood in the ‘No Load’ units. The reason for thick walls around the edge of the pavers may be due to higher stability of the load on top of the pavers, this possibility will require further investigation.

Termites build with clay, presumably because clay with specific particle sizes, together with saliva and faeces, has desirable building properties, such as low shrinkage, water-proofing, water-holding, and high stability^13^,^15^,^33^,^36^. This has been considered most carefully in nest building by termites and ants, the largest and most spectacular insect constructions^13^,^14^,^15^. Two higher termites, *Macrotermes bellicosus* from Africa^37^, and *Cornitermes cumulans* from South America^38^ use clay to reinforce their mound-nests during construction. A leaf-cutting ant, *Atta vollenweideri* from South America^39^, used clay plus sand to create porosity and thus allow ventilation, in contrast the mangrove living *Polyrhachis sokolova* from Australia used sand to block nest entrances and prevent flooding^40^.

If our hypothesis, that adding clay support increases food resource, is correct, then the energy cost of moving clay from the soil to the wood must be lower than the energy gained from eating the wood. For the field experiment, we estimated that the average energy expended by termites moving the clay to make the load bearing clay walls (i.e. clay in the ‘With Load’ units minus clay in the ‘No Load’ units) to be 7.8 J, using measurements on the clay in *C. acinaciformis* mounds in Northern Australia (mound walls are 47% kaolinite, 23% boehmite and 10% gibbsite, which are found at average depths of 22.8 cm, 31.5 cm and 11.0 cm respectively^40^), and assuming the termites use an equal amount of water as clay during building. We estimated the average energy in the wood consumed by the termites to be 5,573 J (assuming average values for fast grown *Pinus radiata*, and 29% indigestible lignin content^41^,^42^,^43^). This represents a 700-fold return on energy expended in clay building, clearly a good foraging strategy.

The termites may have another strategy to optimise foraging; minimising their clay building at any time. In the laboratory experiment the termites ate the unloaded wood first. This wood did not require supportive clay walls; therefore by eating this wood first, the termites prioritised the highest net energy gain. In addition, in both experiments, the termites built their load-supporting clay walls progressively, as they consumed the wood. This progressive construction suggests that the termites built walls that were just strong enough to support the load at that point; they did not build a complete clay wall before consuming the wood. Building a complete wall may be wasted effort, should the termites have to abandon the food before it was consumed, perhaps due to the appearance of predators or superior competitors.

The use of clay to support loads may have evolved from following self-organizational rules arising from responses to single stimuli. These self-organisation rules have been observed in all animals^43^, including other eusocial insects, including ants^44^,^45^,^46^,^47^, bees^48^,^49^,^50^, and also in termites^18^,^19^,^43^,^51^. The building response of *C. acinaciformis* may be triggered by the stimuli of vibrations or acoustic properties of the wood under load, as shown for other termite species^16^,^18^,^51^. A dynamic response may coordinate the two behaviours. First, a termite worker bites the loaded wood, perceives a vibration signal that indicates the wood is under load, and therefore changes behaviour from feeding to transporting clay. After a certain amount of clay has been transported, or after a certain amount of time has elapsed, then the termite worker bites the wood again. If sufficient clay has been added to support the load, the vibrational signal in the wood should have changed sufficiently and so the termite eats the wood. This continues until the vibration signal in the wood indicates it is under load once again. A similar pattern has been described for nest excavation in termites^52^ and ants^53^.

This study raises a question about the role of building with clay in evolutionary success. The clay added by *C. acinaciformis* to support loads unlocks a previously unavailable food resource, which would represent an evolutionary advantage over more basal termites that do have this ability. There are four *Coptotermes* species that build mounds, the only ‘lower termites’ (Family Rhinotermitidae i.e. more phylogenetically basal) to do so^54^; all other mound-building termites belong to the ‘higher termites’ (Family Termitidae)^14^,^15^. *Coptotermes* is the sister genus to the Termitidae, or the higher termites, which are the largest and most successful family of termites^55^,^56^. Perhaps the use of clay as a foraging tool is an important evolutionary step in aboveground mound construction, initially derived from building covered runways to avoid exposure when foraying into the open air. If so, then clay as a foraging tool may be widespread among the family Termitidae.

## Materials and Methods

**Figure 2:**
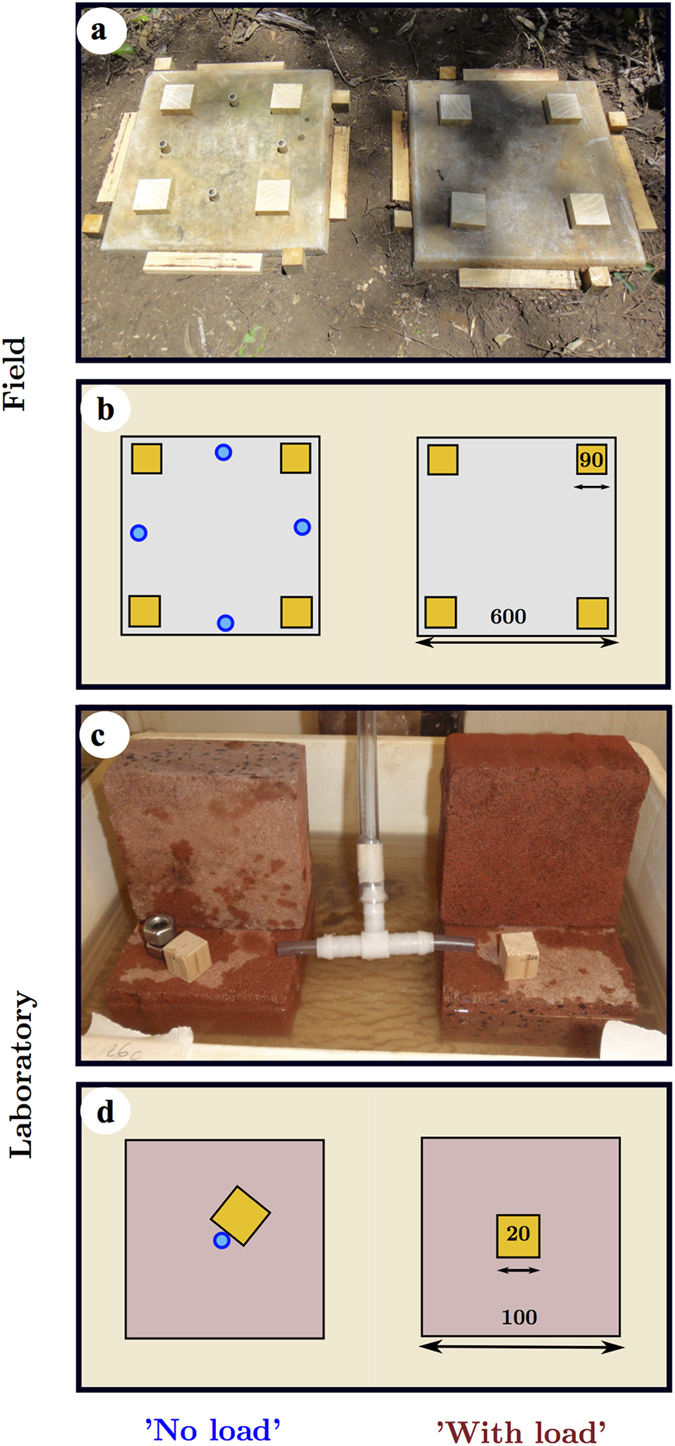
Experimental set-up to test termite ability to distinguish wood under load. The paired experimental units in the field experiment (**a**) photograph and (**b**) schematic; and the laboratory experiment (**c**) photograph and (**d**) schematic. For the schematics, the wood is coloured yellow (small squares) and the metal supports (small circles) holding the load in the ‘No load’ control are coloured blue; all lengths in mm; Photo credits: Sebastian Oberst and Patrick Gleeson (CSIRO, Canberra).

**Figure 3:**
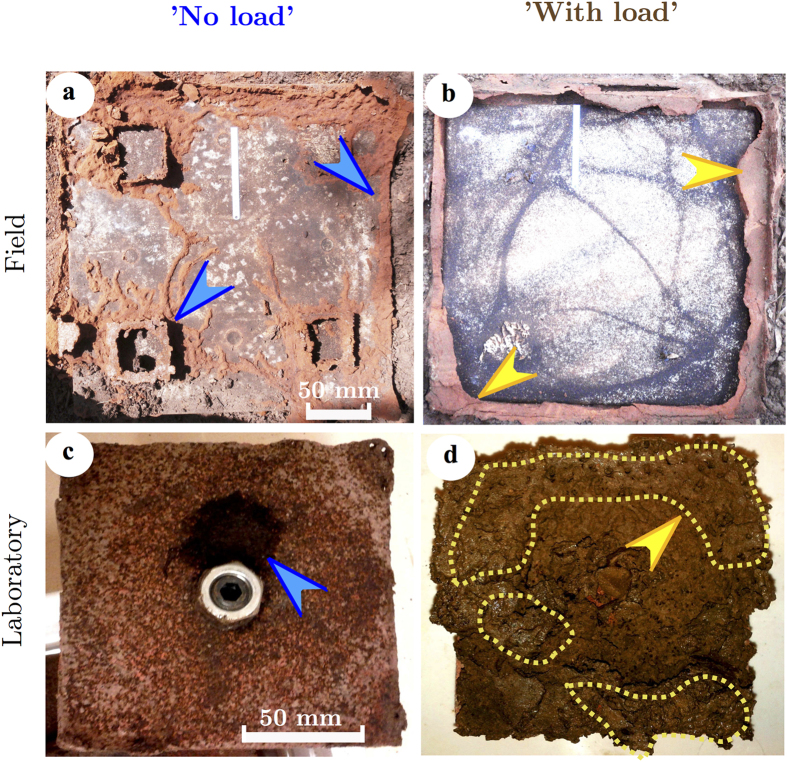
Clay structures built by termites in field and laboratory experiments. Field experiment: (**a**), ‘No load’ units had thin and brittle sheeting, mostly around wooden blocks and often incomplete, for ‘camouflage’; (**b**), ‘With Load’ units had a thick and solid wall, mostly around periphery of concrete paver. Laboratory experiment: (**c**), ‘No load’ units had little mudding, usually positioned beside the wooden block; (**d**), ‘With load’ units had entire paver covered by mud, usually with thicker walls (indicated by yellow dotted line).

**Figure 4:**
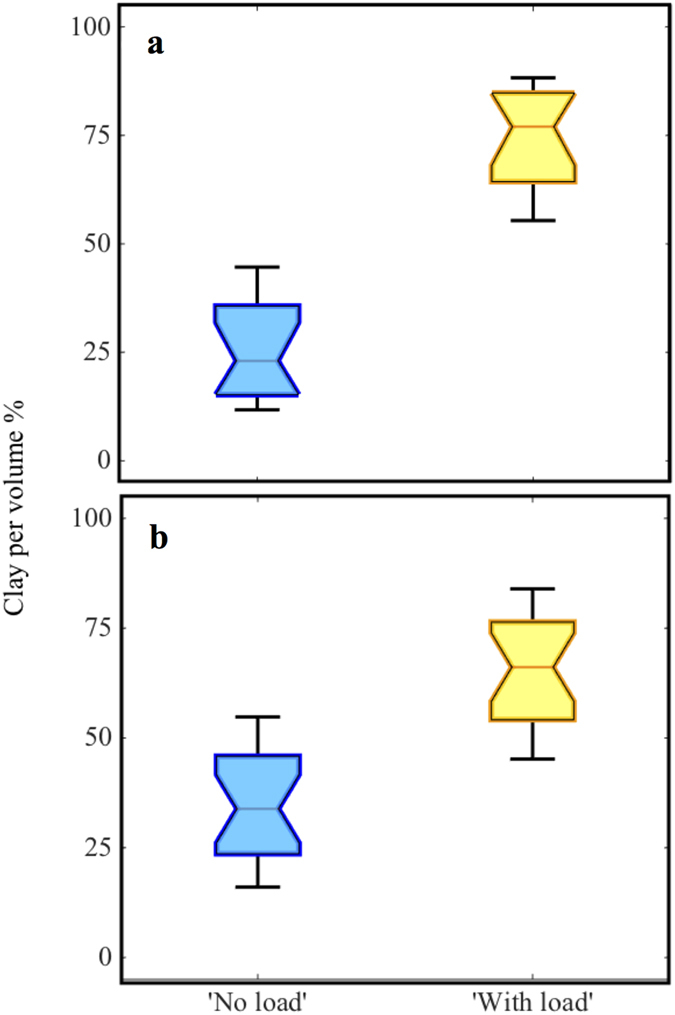
The amount of clay used by termites to build structures in (a) field and (b) laboratory experiments. Quantities are box-plots of clay mass per volume between pavers, central horizontal lines are medians, notches are 95% confidence intervals of the medians (non-overlapping indicates significant differences), boxes are 25th and 75th percentiles, whiskers are the extremes of the distribution.

**Figure 5:**
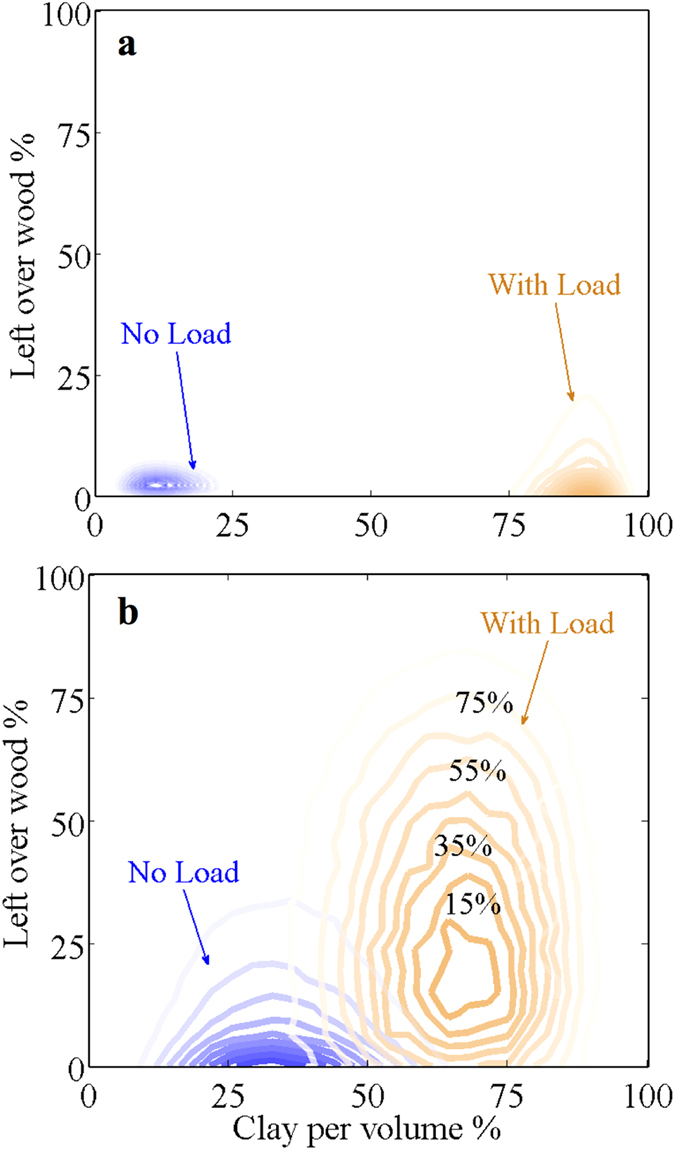
The relationship between clay structures built by termites and left over wood uneaten by termites. **(a)** field experiment and **(b)** the laboratory experiment. The bivariate and normalised joint probability distribution (plotted as relative frequencies, the contours indicate the percentage covered by the distribution) for the ‘With load’ units shows that as clay is added wood is removed. Time may be a factor as no clear ‘line of identity’ (diagonal) is present.

**Figure 6:**
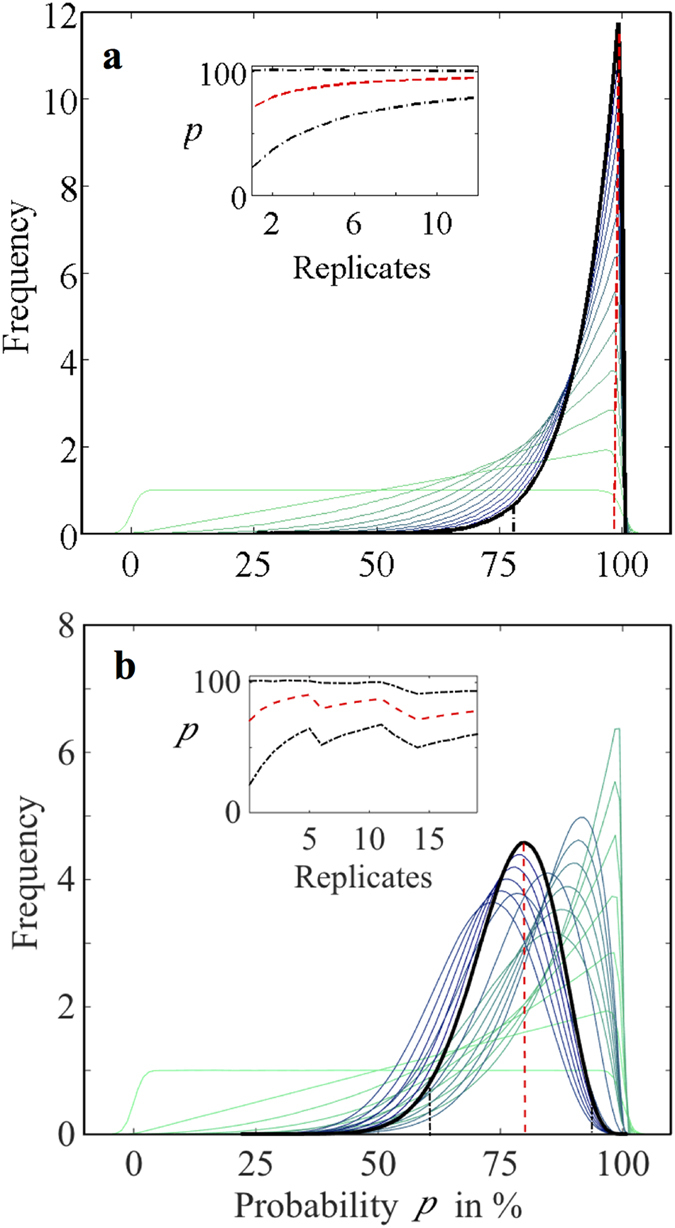
The Bayesian beta-binomial model showing termites substitute wood with clay. for the ‘With Load’ units of **(a)** the field experiment and **(b)** the laboratory experiment. The evolution of the beta-binomial distribution over increasing number of replicates (from light to dark green, then light to dark blue, to black) shows the median probability of termites substituting wood through clay. The insert shows the 99% Credibility Interval at the highest density region

**Figure 7:**
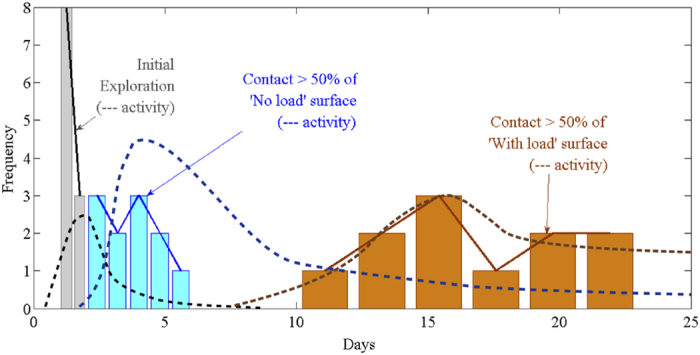
Termite foraging as dynamic process in the laboratory experiment. First termites explored both units, second they fed on the wood in the ‘No load’ unit, and third, they fed on the wood in the ‘With load’ unit. Histograms represent termite contacts (linearly interpolated bin-centres) solid lines connect histogram centres, dashed lines are activity predictions as Kernel estimated normal distributions.

Our basic test unit for field and laboratory experiments comprised wooden blocks (*Pinus radiata)* of 90 × 90 × 30 mm^3^ and 20 × 20 × 20 mm^3^ for the field and the laboratory experiment respectively sandwiched between concrete pavers. Each unit comprised two different parts, which consisted of either a ‘With load’ treatment side or a ‘No load’ controls (all load was carried by metal spacers; Figs 2, S1 and S2).

Wood grain was vertical (i.e. parallel to the load), mimicking that in trees or studs in houses. We measured wood consumption and clay utilised to build wall structures (all oven dried for eight hours at 105 °C, after which weight did not change), and then calculated the relative amount of clay per unit volume between the concrete pavers by disassembling the paver pairs, recovery of the materials by hand, followed by drying to constant weight at 105 °C.

### Field experiment

We conducted the field experiment at the Darwin Laboratory of the Commonwealth Scientific and Industrial Research Organisation (CSIRO) located in Berrimah (Northern Territory; 12° 28′ 52″S, 131° 1′ 44″E). We tested the relative health of *C. acinaciformis* colonies using the mound damage and repair method; 18 colonies of at least 1 km apart were considered to be healthy and used in the experiment. Preliminary heath check were conducted by damaging the mound locally with a 100 × 100 mm^2^ hole; if the mound was fully repaired over night the colony was considered healthy. Figure 2(a,b) and S2 depict the experimental design of the field trial as a bioassay food-choice experiment. Wood as food for the termites was positioned between concrete pavers (45 kg each), which became loaded with water barrels of about 200 L. The mass of the load sitting on top of the wooden blocks mimicked the situation of termites living attacking load bearing timber in houses or the natural situation as found in trees. The termites chose between unstressed (‘No load’, control, load resting on metal spacers) and stressed wood (‘With load’, treatment). The initial wood weights (about 424 ± 22.01 g) were closely matched with an average weight difference between wood pieces of only 1.003 ± 0.012 g. The field experiment considered the total wood consumption and building activities by colonies over a year. We found that the average compressive strength (Instron (ITW), Norwood, Massachusetts, USW, 8033 servo- hydraulic compression testing machine, 5 kN load cell rating) of undamaged clay wall samples was about 0.22 MPa (*n* = 3). The clay walls built around the pavers in the field experiment had a cross sectional area of around 236 cm^2^, which could support over 5236 N, or 536 kg – more than enough to support the 245 kg load imposed on the four wood blocks.

### Laboratory experiment

We conducted the laboratory experiment at the ‘Black Mountain Laboratories’ of the Commonwealth Scientific and Industrial Research Organisation (CSIRO) in Canberra (Australian Capital Territory; 35° 16′ 29.47″ S, 149° 6′ 51.32″E). We collected a total of ≈ 400,000 *C. acinaciformis* termites from three mounds near to Darwin (CSIRO special research permit). We placed 35 g (≈ 8,140 individuals) of termites into 1 L plastic jar, along with 500 g of water soaked mound material and about 300 g wood, and then transported the jars to Canberra. In the laboratory, we connected the 1 L plastic jars containing the termites to a 2 L glass jar containing dried and powdered clay collected from *C. acinaciformis* mounds, and to the experimental units, using plastic tubing (Fig. 2(c,d) and S2). The termites were provided with ground mound material of the outer wall as a building material and the paver pairs placed in a water tray to prevent the termites from escaping. The ‘With load’ units were only loaded with the deadweight of a single paver in order to analyse whether significant qualitative differences to the results in the field were found. The dead weight here simulated therefore a lower bound of termites to distinguish between the unloaded and loaded side, a situation that might be encountered in smaller trees or branches. Visually inspecting the clay building activity on the ‘No load’ and the ‘With load’ units every day for about four weeks tested the activity of the termites in the laboratory experiment. All laboratory experiments were conducted in conditioned rooms with about 28 °C and 80% relative humidity using 32 groups of termites. The initial wood weights (about 3.32 ± 0.20 g) were closely matched with an average weight difference between wood pieces of only 0.012 ± 0.008 g). The laboratory experiment investigated groups of termites’ partial wood consumption and building as related to dynamic behaviours under controlled conditions over four weeks.

### Data analyses

We tested the hypotheses using balanced one-way analysis of variance (ANOVA) tests on the absolute amount of clay, the relative amount of clay per setup (as in the ‘With load’ units the separation of the pavers may reduce as the wood is consumed) and the relative amount per setup normalised by the volume underneath the concrete pavers in order to show on which unit (unloaded or loaded) termites utilise more building material. In order to determine the influence of wood on the building behaviour we calculated two-sample Kolmogorov-Smirnoff tests to test if the field and the laboratory experiment originate from the same distributions. Then we fitted the data to theoretical continuous marginal beta-distributions and produced predictive bi-variate joint distributions of relative remaining wood and relative clay per unit volume by drawing 10000 bootstrapped samples. We determined directly the probability of termites utilising clay as a building tool by taking the reduction of wood as conditional to the building of clay and applied Bayesian inference to a beta-binomial model (uni-variate distribution as Jeffrey’s prior^28^). As software for statistical data analysis and plotting we used MatLab 2013b and R x64 2.13 with R Studio 0.97.332.

## Additional Information

**How to cite this article**: Oberst, S. *et al.* Termites utilise clay to build structural supports and so increase foraging resources. *Sci. Rep.* **6**, 20990; doi: 10.1038/srep20990 (2016).

## Supplementary Material

Supplementary Information

## Figures and Tables

**Figure 1:**
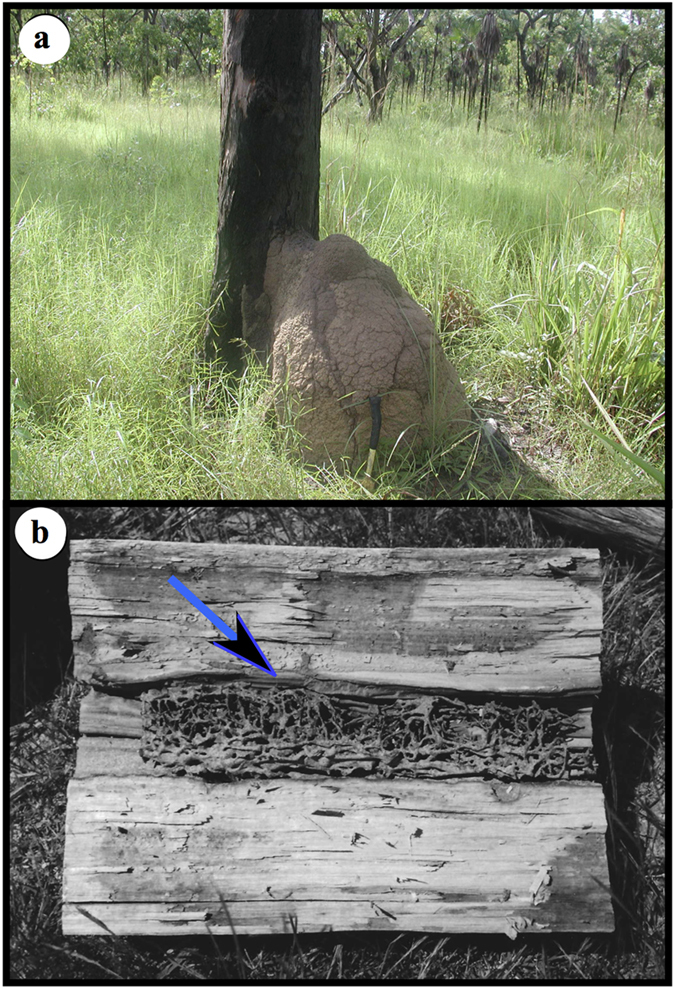
Nesting and foraging of *Coptotermes acinaciformis*. **(a)** The mound nest leaning against infested tree (tropical north of Australia), **(b)** telegraph pole hollowed out by *C. acinaciformis*; Photo credits CSIRO, Canberra.
