# Supplementary material for: Termites utilise clay to build structural supports and so increase foraging resources
Source: Sci Rep. 2016 Feb 8;6:20990. doi: 10.1038/srep20990 (PMC4745099; doi:10.1038/srep20990)
Supplement: Supplementary Information [file srep20990-s1.pdf]

Supplementary Information

Termites utilise clay to build structural supports and so increase foraging resources

Sebastian Oberst*[1, 2], Joseph C.S. Lai[1], Theodore A. Evans[1, 3]

[1]Acoustics & Vibration Unit, School of Engineering and Information Technology, The University of New South Wales, ADFA, Canberra, ACT 2600, Australia

[2]CSIRO Ecosystem Sciences, Clunies Ross Street, Canberra ACT 2600, Australia

[3]School of Animal Biology, University of Western Australia, Perth, WA, 6009, Australia

* For correspondence email Sebastian Oberst (email: s.oberst@adfa.edu.au)

Background Information. *Coptotermes acinaciformis* is a major pest species and often found infesting houses and other timber in service[1]. The legendary 'foot through the floor board' has been reported in little stressed wood partitions often associated with large amounts of clay[2,3] with termites having often already left the foraging site. Figure S1 exemplifies damage caused by *C. acinaciformis*, which only became visible after removing the retaining walls: while in Figure S1(a) the left wooden stud has been significantly reduced, the studs in Figure S1(b) show that the damage is accompanied by clay building activity (partially collapsed owing to retaining wall removal).

[Figure S1 about here]

Experimental Setup. The principle of setting up the field and the laboratory experiment are detailed in Figures S2 and S3. After placing wood and metal spacers on a concrete paver close to a termite mound, the setup was covered with a second paver, a plastic cover and soil, and then a load was applied to the 'With load' unit (Figure S2). In the laboratory experiment one jar with a group of termites was connected to a jar with ground termite mound material which led over plastic tubing to the 'Without load' and 'With load' pavers, surrounded by water to avoid the termites from escaping (Figure S3).

[Figure S2 about here]

Bi-variate distribution. The beta distribution's location (a) and the scale (s) parameters of the 'relative amount of clay/ volume', of the field experiment are a = 8.352 & s = 43.863 ('No load') and a = 52.215 & s = - 43.863 ('With load'). For the variable "remaining wood" these parameters are a = 2.553 & s = -1.222 ('No load') and a = 1.331 & s = 1.222 ('With load'). For the laboratory experiment's 'relative clay / volume' the parameters are a = 5.257 & s = 4.401 ('No load') and a = 9.658, s = - 4.401 ('With load') and for the remaining wood a = 0.481 & s = 0.391 ('No load') and a = 0.872 & s = -0.391 ('With load').
Analysing the experiments' bi-variate distributions showed that the joint distribution for the laboratory experiment is flatter than the field trial especially on the control unit indicating a higher variability than the 'No load' unit. These differences in sharpness (from rather platykurtic to leptokurtic) of the distribution could be attributed to the different runtimes used for the experiment and the behaviour of termites firstly to have the tendency to dwell on the control unit and consume its wood, then to move on to reduce the wood on the treatment unit accompanied by strong mudding activity.

[Figure S3 about here]

Beta-binomial model. By assuming a beta-binomial distribution *(4) beta(*| s, f)* (s successes, f failures, success defined of having >50 % of total clay / volume as well as having the ≥ 50 % of the total remaining wood on the 'With load' unit) and applying a continuous kernel estimate, we calculated and visualized the sequence of successes and failures approximated by continuous beta distributions. As parameters for the kernel estimate we used 10 million iterations with a step size of 0.001 to approximate the tails of the distribution which otherwise overlap the theoretically valid interval [0, 1] more for less iterations with coarser step size. For n = 0 a uniform distribution was approximated[4] using a so-called initial or Jeffrey's prior. Jeffrey's prior is used here as compared to a 'qualified prior' [5], which shows a quicker convergence but also implicitly higher uncertainty as based on experiments.
For n = 1 a linearly increasing function was approximated with its maximum at $p \sim 1.0$ resulting in narrower distribution curves which became centred around the true value as more information came in. With the beta-binomial model the unknown median probability p of a binomial model was estimated; its credible interval as highest density region (HDR) provided a 95% confidence interval[5]. Figure 6 (main document) shows the beta-binomial distribution and their 95 % credible bounds (HDR:= highest density region) as inserts for the median probability of having more mud on the

treatment unit. The probability is far higher than 50 % (the trivial solution) and with 95 % confidence 99.2 %, HDR = [78.7, 100] % and 79.2 %, HDR = 60.2, 93.5] % for the field and the laboratory experiment. Also here we observed more platykurtic distributions for the laboratory experiment than for the field trial indicating its higher variability and probably the character of laboratory experiments of only being approximate for the field trials.

Termite activity level. For the laboratory experiment, wood and clay per unit volume (both in %) were uncorrelated for the 'No load' unit (Pearson correlation coefficient ρ = 0.02) and moderately negative correlated for the loaded unit (ρ = - 0.38). In the laboratory experiment it was possible to monitor, which unit was contacted first and where building activity was higher as the sides of pavers were left open. In 11/20 setups it could be observed that the termites equally explored both the treatment and the control unit as a clay trail was leading to the wood block (Figure 7, main document). In 81.8 % of the sets the control unit was contacted heavily after three days (covering more than 50 % of the exposed surface area) while the treatment unit was not further mudded in. However, after a runtime of about two weeks, more clay was build up on the treatment unit (more than 50% of the exposed areas covered by then) while the wood on the control and treatment unit was on average reduced by 75 % and 65 %. The height of the treatment unit was in the end on average 68 ± 22 % of the initial height, which correlated, moderately positive with the left over wood (ρ = 0.50).

References Supporting Information

1. Andersen, P. Jacklyn, *Termites of the Top End.* CSIRO Australia. 1993.
2. G.R. Sandlant, *Coptotermes acinaciformis* (Froggatt), *Coptotermes frenchi* (Hill) (Isoptera: Rhinotermitidae): Australian subterranean termites in New Zealand. Wellington: Forest Research Institute. 1985
3. T.A. Evans, P.V. Gleeson, The effect of bait design on bait consumption in termites. *Bull Ent Res*, **96**, 85–90(2006).
4. A. Spanos, Probability Theory and Statistical Inference. Cambridge University Press, UK.1999.
5. S.A. Schmitt, Measuring Uncertainty. Addison-Wesley Publishing Company: Reading, Massachusetts - Menlo Park, California - London - Don Mills, Ontario. 1969.

Figure Legends Supplementary Information

Figure S1. Illustration of termite activity.
Termite infestation (*Schedorhinotermes intermedius*) of internal plaster wall with timber frame located between bedroom and bathroom (Sydney, NSW, Australia); **(a)** Termite attack of unloaded structure, wood reduced with almost no clay present (arrows) only the second stud to the right is still bearing load; and **(b)** loaded structure accompanied by provision of large amounts of solid materialised clay soils indicated by arrows (partly collapsed owing to being dried out and shrinkage of the clay); photo credits Patrick Gleeson, CSIRO, Canberra.

Figure S2. Field experimental setup.
(a) Concrete pavers with aluminium studs (left setup, 'No Load') and wood blocks; **(b)** second concrete paver; **(c)** plastic sheet and weight on treatment unit ('With load', right setup); and **(d)** covering up with a layer of soil; photo credits Patrick Gleeson, CSIRO, Canberra.

Figure S3. Laboratory experimental setup.
(a) Photograph showing laboratory setups, with paired pavers (dead weight as load), termite jars and clay jars, connected with plastic tubing; **(b)** range of setups sitting in a shelf of the laboratory after about a week.

Figures Supplementary Information

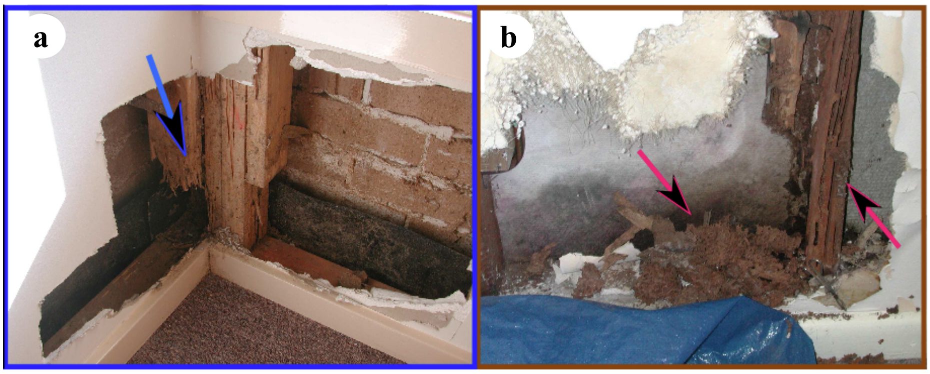

Figure S1.

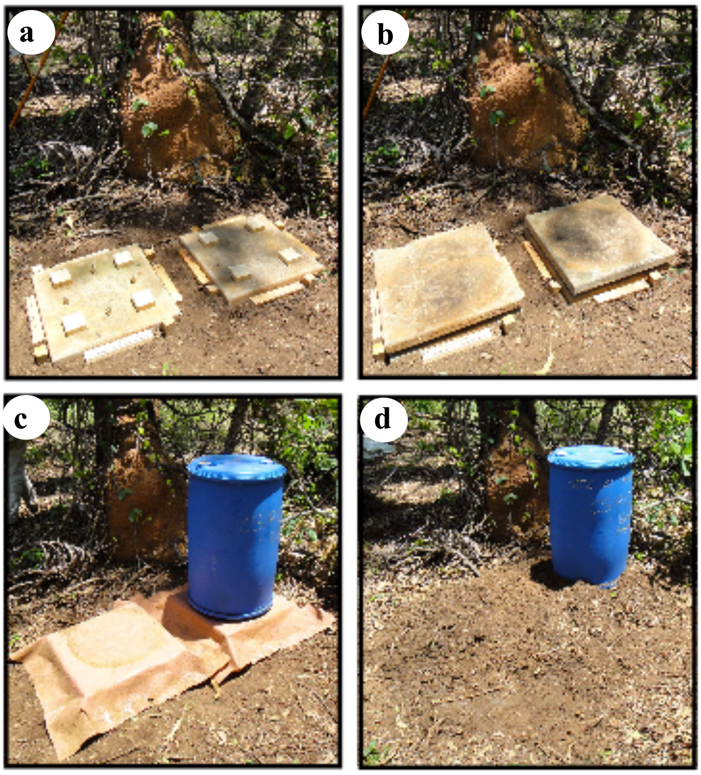

Figure S2.

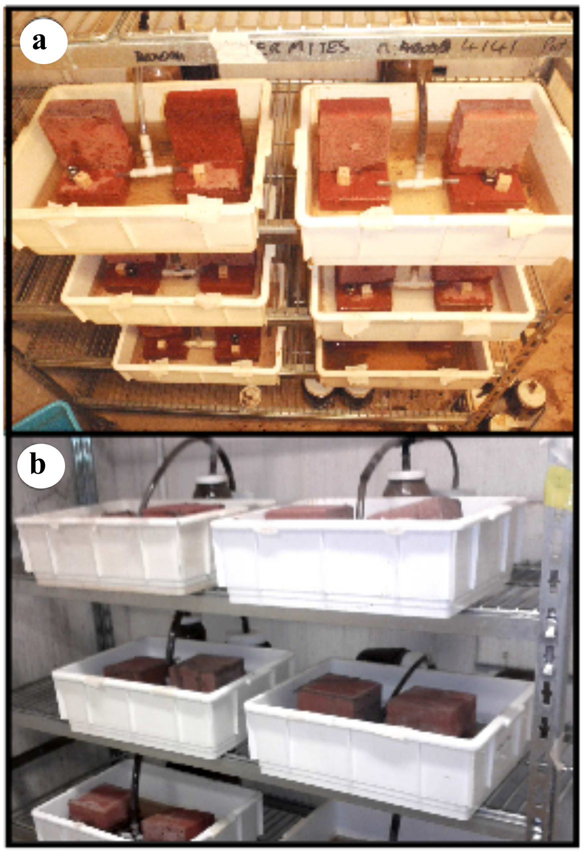

Figure S3.
